# Unmet needs in palliative care for patients with common non-cancer diseases: a cross-sectional study

**DOI:** 10.1186/s12904-022-01040-0

**Published:** 2022-08-30

**Authors:** Hyoeun Jang, Kyunghwa Lee, Sookyung Kim, Sanghee Kim

**Affiliations:** 1 College of Nursing and Brain Korea 21 FOUR Project, Yonsei University, 50-1, Yonsei-ro, Seodaemun-gu, 03722 Seoul, Republic of Korea; 2 College of Nursing, Konyang University, 158, Gwanjeodong-ro, Seo-gu, 35365 Daejeon, Republic of Korea; 3 School of Nursing, Soonchunhyang University, 50, Suncheonhyang 4-gil, Dongnam-gu, Chungcheongnam-do 31151 Cheonan-si, Republic of Korea; 4 College of Nursing and Mo-Im Kim Nursing Research Institute, Yonsei University, 50-1, Yonsei-ro, Seodaemun-gu, 03722 Seoul, Republic of Korea

**Keywords:** Palliative care, Needs assessment, Anxiety, Depression, Quality of life, Heart failure, Stroke, Kidney failure, Chronic, End-stage liver disease

## Abstract

**Background:**

Non-cancer patients experience the chronic process of disease that increases the patients’ suffering as well as families’ care burden. Although two-thirds of deaths are caused by non-cancer diseases, there is a lack of studies on palliative care for non-cancer patients. This study identified the palliative care needs and satisfaction, anxiety and depression, and health-related quality of life (HRQOL) of non-cancer patients and identified the factors influencing their HRQOL.

**Methods:**

A cross-sectional survey design was employed. Participants were 114 non-cancer patients with chronic heart failure, stroke, end-stage renal disease, or end-stage liver disease who were admitted to the general ward of a tertiary hospital in South Korea. Measures included the Palliative Care Needs and Satisfaction Scale, the Hospital Anxiety and Depression Scale, and the Medical Outcome Study 36-items Short Form Health Survey version 2. Data were analysed with descriptive statistics, independent *t*-tests, analyses of variance, Pearson’s correlations, and multiple linear regression analyses.

**Results:**

The average score of palliative care needs was 3.66 ± 0.62, which falls between ‘moderate’ and ‘necessary’. Among the four domains, the average score of palliative care needs in the psychosocial domain was the highest: 3.83 ± 0.67. Anxiety was nearly in the normal range (7.48 ± 3.60; normal range = 0–7) but depression was higher than normal (9.17 ± 3.71; normal range = 0–7). Similar to patients with cancer, physical HRQOL (38.89 ± 8.69) and mental HRQOL (40.43 ± 11.19) were about 80% of the general population’s score (50 points). Duration of disease and physical performance were significant factors associated with physical HRQOL, whereas physical performance, anxiety, and depression were significant factors associated with mental HRQOL.

**Conclusion:**

It is necessary to maintain non-cancer patients’ physical performance and assess and manage their mental health in advance for effective palliative care. This study provides relevant information that can be used to develop a tailored palliative care model for non-cancer patients.

## Background

Palliative care is an approach to improving the quality of life (QOL) of patients and their families facing health problems owing to life-threatening diseases. It prevents and alleviates suffering using physical, mental, social, and spiritual approaches [1]. The World Health Organization has suggested, in addition to cancer, non-cancer diseases should be subject to palliative care [2]. Given the changing climate around palliative care, the South Korean government implemented the Act on Hospice and Palliative Care and Decisions on Life-Sustaining Treatment for Patients at the End of Life in 2016. The Act expanded the scope of palliative care to include patients with chronic liver cirrhosis, chronic obstructive respiratory disease (COPD), and AIDS, in addition to cancer [3]. Although about 70% of people died from non-cancer diseases such as cardio-cerebrovascular, liver, or lower respiratory tract diseases in 2019 [4], 99.9% of new users who utilised the palliative care service in 2019 were patients with cancer [5]. Since the existing palliative care was developed for patients with terminal cancer [6], the cancer patient-based palliative care model is likely inappropriate to meet the needs of non-cancer patients [7]. Therefore, a palliative care model that considers the characteristics of different non-cancer patients and their palliative care needs is required.

A previous study comparing the palliative care needs of patients with cancer and common non-cancer diseases found they had a similar prevalence of physical, psychological, social, and spiritual concerns [8]. However, patients with common non-cancer diseases such as COPD, end-stage renal disease (ESRD), and heart failure had less function and were less likely to receive palliative care than patients with cancer [9]. Patients with cardiopulmonary failures were less likely to use palliative care than patients with cancer but had a higher risk of being hospitalised in an intensive care unit or receiving life-sustaining treatment [10, 11]. This suggests that the low use of and late referral to palliative care are owing to a lack of knowledge about the needs of non-cancer patients [6]. Although these previous studies [9–11] tried to identify the palliative care needs of non-cancer patients, they mainly focused on physical symptoms, making it difficult to determine palliative care needs from an integrated perspective. Given that non-cancer patients have a similar symptom burden but heterogeneous trajectories compared to cancer patients [11, 12], we should consider that the palliative care needs of non-cancer patients will differ from those of patients with cancer [11]. Therefore, it is important to identify their specific needs to provide palliative care suitable for non-cancer patients.

Patients’ satisfaction—which is essential for evaluating the quality of palliative care [13]—allows us to understand the present condition of the palliative care that is provided to patients and how it can be improved. Concerning palliative care satisfaction, studies have mostly focused on caregivers or bereaved families. In previous studies, it was reported that caregivers had high satisfaction with the palliative care provided to patients [14–16]. However, the overall satisfaction was evaluated without considering the characteristics of palliative care that pursues an integrated approach. To our knowledge, studies on palliative care satisfaction in non-cancer patients have been difficult to find. In a study targeting patients with cancer, satisfaction was high before and after provision of palliative care [17]. Since this evaluated palliative care satisfaction related to communication with medical staff and system functions of healthcare services, there was a limitation in that it was difficult to understand in detail which part of the care was satisfied or dissatisfied. In oncology, the FAMCARE-Patient scale—developed based on the FAMCARE scale, a tool developed for patients’ family—measures patients’ palliative care satisfaction. However, the FAMCARE-Patient scale has limitations in evaluating satisfaction in psychosocial and spiritual domains, which are important in palliative care, because it consists of questions about care for physical symptoms, provision of information, medical staff, etc. [18]. Considering that non-cancer patients’ use of palliative care is very low in reality, it is expected that even patients who are hospitalised in a general unit rather than a palliative care unit will receive care in the context of palliative care. Therefore, to provide high-quality palliative care, it is meaningful to understand palliative care satisfaction based on the care they are currently receiving.

Patients suffer due to various symptoms, however, psychological issues are likely to be overlooked because providing care for physical symptoms is sometimes prioritized [19]. The most frequently reported psychological problems among patients receiving palliative care were anxiety and depression [20], which decreased the QOL of non-cancer patients as well as patients with cancer [21]. Although there are similarities in the patterns of health problems between non-cancer and patients with cancer [22], it is inappropriate to equate the approach to palliative care. It remains necessary to understand if non-cancer patients have specific psychological health problems that patients with cancer do not have [12]. Detailed identification of their psychological problems can help expand our understanding of how best to meet their palliative care needs [23].

Studies on the anxiety, depression, and QOL of patients with cancer in relation to palliative care have been continuing in recent years [24–29]. Previous studies on non-cancer patients only suggest the need to provide palliative care to reduce anxiety and depression and improve their QOL. However, there are insufficient studies specifically identifying their levels of anxiety, depression, and QOL. Hence, assessment of anxiety, depression, and QOL in non-cancer patients is essential to provide them with suitable palliative care. This study will contribute to the preparation of basic data for the development of a suitable palliative care model for non-cancer patients.

## Methods

### Study aims

This study aimed to (1) examine palliative care needs and satisfaction, anxiety and depression, and health-related QOL (HRQOL) in patients with common non-cancer diseases; and (2) identify factors that influence their HRQOL.

### Study design

This study used a cross-sectional descriptive design.

### Participants

The study employed convenience sampling of patients with non-cancer diseases such as chronic heart failure (CHF), stroke, ESRD, or end-stage liver disease (ESLD) who were hospitalised at a tertiary hospital in Seoul, Korea. The inclusion criteria were: (1) adults hospitalised for conservative treatment with CHF, stroke, ESRD, or ESLD; and (2) ability to communicate and voluntary agreement to participate in the study. Exclusion criteria were patients who were hospitalised for organ transplantation and patients registered on the waiting list for transplantation.

The sample size was calculated for a multiple linear regression analysis, a medium effect size (Cohen’s *f*^*2*^) of 0.15, two-tailed significance of 0.05, power of 80%, and nine predictors using G*power version 3.1.9.2 [30]. The minimum sample size required was 114 participants, and 127 questionnaires were distributed in consideration of a 10% dropout rate. A total of 114 questionnaires were finally analysed after 13 questionnaires with insufficient responses were excluded.

### Data collection

 We recruited participants from December 5, 2017 to June 10, 2019 at Severance Hospital of Yonsei University, Korea. With the cooperation of doctors and unit managers, researchers visited the ward to promote recruitment. The researchers fully explained the study to potential participants and encouraged them to make voluntary decisions about their participation. After confirming that participants fully understood, the researchers obtained informed written consent. The questionnaire was completed face-to-face by trained researchers and took about 20 min.

### Measures

#### Demographic and clinical characteristics

We assessed participants’ demographic characteristics such as age, sex, marital status, educational level, primary caregiver, employment status, family monthly income, religion, and hospice experience. Clinical characteristics included disease type and severity, hospitalisation period, number of comorbidities, duration of illness, and Eastern Cooperative Oncology Group Performance Status (ECOG PS) [31].

The ECOG PS is graded as follows: Grade 0, fully active, able to carry on all pre-disease performance without restriction; Grade 1, restricted in physically strenuous activity but ambulatory and able to carry out work of a light or sedentary nature; Grade 2, ambulatory and capable of all self-care but unable to carry out any work activities, up and about more than 50% of waking hours; Grade 3, capable of only limited self-care, totally confined to bed of chair; Grade 4, completely disabled, unable to carry on any self-care, and Grade 5, dead.

#### Palliative care needs and satisfaction

Palliative care needs and satisfaction were measured with the Hospice Nursing Needs and Satisfaction Scale modified by Kim et al. [32] based on the scales developed by Jang [33] and Kang and Kim [34]. We selected this scale because it can identify needs and satisfaction in detail by domain in the integrated aspect of palliative care. The scale contains 37 items across four domains: physical (10 items), psychosocial (10 items), spiritual (8 items), and educational and referral (9 items). Examples of items are as follows: ‘care for pain management’, ‘care for symptoms and fatigue’ in the physical domain; ‘care for listening to the patients’ complaints’, ‘care for encouraging emotional stability and empowerment’ in the psychosocial domain; ‘care for respecting religion’, ‘care for being helped by religion’ in the spiritual domain; and ‘connecting doctors, volunteers, social workers, or nutritionists’, ‘nursing education for preparing for death’ in the educational/referral domain. Answers were scored on a 5-point Likert scale ranging from 1 (*very unnecessary*) to 5 (*very necessary*) for palliative care needs, and from 1 (*very dissatisfied*) to 5 (*very satisfied*) for palliative care satisfaction. The higher the mean scores, the higher the levels of palliative care needs and satisfaction. This scale was initially developed for patients with cancer and its content validity was verified by six experts. Lee et al. [35] utilised this scale, after verifying its content validity, to identify the unmet needs of patients with Parkinson’s disease. In the original study [32], Cronbach’s alphas were 0.93 for needs and 0.91 for satisfaction. In this study, Cronbach’s alphas were 0.95 for both, indicating good reliability.

#### Anxiety and depression

Anxiety and depression were measured with the Hospital Anxiety and Depression Scale (HADS) by Zigmond and Snaith [36], purchased from the GL Assessment website [37]. Anxiety and depression are common mental health problems. This scale is a widely used, validated and reliable scale to measure mental health-related problems in patients with cancer and various non-cancer diseases [38]. As a scale commonly used in cancer patient research, it is considered appropriate for comparison with the status of non-cancer patients. The HADS scale has 14 items in total (seven items in each subscale of anxiety and depression). The scale employs a 4-point Likert scale (0–3); each subscale’s total score is summed from the scores of the individual items. The total scores in each subscale range from 0 to 21. Higher scores indicate higher levels of anxiety and depression. Scores of 0–7, 8–10, and 11–21 indicate normal, borderline, and abnormal cases, respectively. Cronbach’s alphas were 0.89 for the anxiety subscale and 0.86 for the depression subscale in the study that developed the Korean version [39]. In this study, Cronbach’s alphas were 0.73 for anxiety and 0.64 for depression; however, the overall scale has acceptable reliability (α = 0.76).

#### Health-related quality of life

HRQOL was measured using the Korean version of the Medical Outcome Study 36-items Short Form Health Survey (MOS SF-36) (version 2) [40]. It is a valid tool widely used to measure generic HRQOL, regardless of the type of disease from the general population to the patient population. MOS SF-36 comprises 36 items across eight scales that measure the following domains of HRQOL: physical functioning, role-physical (changes to usual role activities owing to physical difficulties), bodily pain, general health, vitality, social functioning, role-emotional (changes to usual role activities owing to emotional difficulties), and mental health. The eight scales are hypothesised from two dimensions: the Physical Component Summary (PCS) and the Mental Component Summary (MCS). Response data were calculated using norm-based scoring (NBS) conducted on Health Outcome Scoring Software 5.1 (Quality Metric, Lincoln, RI, USA). NBS yields a distribution of scores in which scores under 50 indicate poorer HRQOL compared to the general population and higher scores indicate a higher level of HRQOL. The Cronbach’s alpha for the original version of the MOS SF-36 was 0.70. In this study, it was 0.68, indicating acceptable reliability.

### Data analysis

We conducted data analysis using SPSS version 26.0 (IBM, Armonk, NY, USA). Descriptive statistics were used to describe the characteristics of participants and their levels of palliative care needs and satisfaction, anxiety and depression, and HRQOL. Independent *t*-tests and analyses of variance were used to examine the difference in HRQOL according to participants’ demographic and clinical characteristics, and we used Scheffé tests to determine whether differences among disease groups. Pearson’s correlation analyses were used to examine the associations between the five main variables. Multiple linear regression analyses were conducted to identify the factors influencing HRQOL.

### Ethical considerations

 We obtained ethical approval from Yonsei University Health System’s Institutional Review Board (no. 4-2017-0615). Informed written consent was obtained from all participants before study commencement. Researchers explained that participants were free to withdraw from the study at any time and that all the information obtained would be managed to preserve anonymity and confidentiality.

## Results

### Participants’ characteristics

Participants’ characteristics are shown in Table 1. Their mean age was 58.42 ± 15.59 years, 46 participants (40.4%) were aged 65 years or older, 59.6% were men, 35.1% had a college-level education or higher, the primary caregiver of 55.3% of participants was their spouse, 52.6% of participants were employed, and four participants had experience with hospice services. Their average hospitalisation period was 7.98 ± 8.80 days, and 33.3% had an illness duration of more than 12 months. In the case of ECOG PS, which indicates their level of physical activity, Grades 0 and 1 were the most common (59.6%).

**Table 1:** Participants’ characteristics (*N* = 114)

Disease type	Total (*N* = 114)	CHF (*n* = 35)	Stroke (*n* = 21)	ESRD (*n* = 28)	ESLD (*n* = 30)
Variables	*M* ± *SD*/*n* (%)	*M* ± *SD*/*n* (%)	*M* ± *SD*/*n* (%)	*M* ± *SD*/*n* (%)	*M* ± *SD*/*n* (%)
Demographic characteristics					
Age (years)	58.42 ± 15.59	61.94 ± 14.33	57.95 ± 16.50	59.29 ± 19.61	53.83 ± 11.09
< 65	68 (59.6)	18 (51.4)	12 (57.1)	14 (40.0)	24 (80.0)
≥ 65	46 (40.4)	17 (48.6)	9 (42.9)	14 (50.0)	6 (20.0)
Sex					
Male	68 (59.6)	21 (60.0)	17 (81.0)	16 (57.1)	14 (46.7)
Female	46 (40.4)	14 (40.0)	4 (19)	12 (42.9)	16 (53.3)
Spouse					
Yes	86 (75.4)	31 (88.6)	17 (81.0)	18 (64.3)	20 (66.7)
No	28 (24.6)	4 (11.4)	4 (19.0)	10 (35.7)	10 (35.7)
Educational status					
≤ Middle school	33 (28.9)	13 (37.1)	4 (19.0)	9 (32.1)	7 (23.3)
High school	41 (36.0)	11 (31.4)	9 (42.9)	7 (25.0)	14 (46.7)
≥ College	40 (35.1)	11 (31.4)	8 (38.1)	12 (42.9)	9 (30.0)
Primary caregiver					
Spouse	63 (55.3)	20 (57.1)	11 (52.4)	16 (57.1)	16 (53.3)
Parents	14 (12.3)	2 (5.7)	3 (14.3)	4 (14.3)	5 (16.7)
Child(ren)	23 (20.2)	9 (25.7)	4 (19.0)	3 (10.7)	7 (23.3)
Other	14 (12.3)	4 (11.4)	3 (14.3)	5 (17.9)	2 (6.7)
Working status					
Unemployed	54 (47.4)	20 (57.1)	6 (28.6)	18 (64.3)	10 (33.3)
Employed	60 (52.6)	15 (42.9)	15 (71.4)	10 (35.7)	20 (66.7)
Family monthly income (USD)					
< 3500	73 (64.0)	23 (65.7)	11 (52.4)	21 (75.0)	18 (60.0)
≥ 3500	41 (36.0)	12 (34.3)	10 (47.6)	7 (25.0)	12 (40.0)
Religion					
None	41 (36.0)	11 (31.4)	8 (38.1)	13 (46.4)	9 (30.0)
Catholicism/Christianity	41 (36.0)	11 (31.4)	7 (33.3)	9 (32.1)	14 (46.7)
Buddhism	32 (28.1)	13 (37.1)	6 (28.6)	6 (21.4)	7 (23.3)
Hospice experience					
Yes	4 (3.5)	0 (0.0)	1 (4.8)	1 (3.6)	2 (6.7)
No	110 (96.5)	35 (100.0)	20 (96.4)	27 (93.4)	28 (93.3)
Clinical characteristics					
Hospitalisation period (days)	7.98 ± 8.80	7.57 ± 7.64	6.00 ± 3.07	8.25 ± 8.62	9.60 ± 12.33
< 7 days	69 (60.5)	22 (62.9)	14 (66.7)	15 (53.6)	18 (60.0)
≥ 7 days	45 (39.5)	13 (37.1)	7 (33.3)	13 (46.4)	12 (40.0)
Number of comorbidities	3.14 ± 1.98	3.94 ± 2.56	1.67 ± 1.24	3.21 ± 1.34	3.17 ± 1.58
≤ 3	74 (64.9)	16 (45.7)	21 (100.0)	16 (57.1)	21 (70.0)
> 3	40 (35.1)	19 (54.3)	0 (0.0)	12 (42.9)	9 (30.0)
Duration of disease (months)					
< 12	76 (66.7)	19 (54.3)	20 (95.2)	22 (78.6)	15 (50.0)
≥ 12	38 (33.3)	16 (45.7)	1 (4.8)	6 (21.4)	15 (50.0)
ECOG PS					
0, 1	68 (59.6)	18 (51.4)	13 (62.0)	13 (46.4)	24 (80.0)
2	23 (20.2)	12 (34.3)	4 (19.0)	3 (10.7)	4 (13.3)
3, 4	23 (20.2)	5 (14.3)	4 (19.0)	12 (42.9)	2 (6.7)

*M* mean, *SD* standard deviation, *CHF* chronic heart failure, *ESRD* end-stage renal disease, *ESLD* end-stage liver disease, *ECOG PS* Eastern Cooperative Oncology Group Performance Status

### Palliative care needs and palliative care satisfaction

Table 2 shows the levels of participants’ palliative care needs and satisfaction. The total score of palliative care needs was 3.66 ± 0.62. By domain, their scores were 3.83 ± 0.67, 3.81 ± 0.73, 3.70 ± 0.72, and 3.23 ± 0.88 in the psychosocial, educational/referral, physical, and spiritual domains, respectively. Among the four disease groups, the CHF group had the highest total score with 3.84 ± 0.67, and their score in the physical domain was significantly higher than the stroke group (*F* = 3.114, *p* = .029). The total score for palliative care satisfaction was similar to that of palliative care needs at 3.60 ± 0.56. The physical and psychosocial domains were the highest, with 3.46 ± 0.65 and 3.44 ± 0.71, respectively; while educational/referral and spiritual domains were lower: 2.97 ± 0.80 and 2.85 ± 0.90, respectively.

**Table 2:** Level of palliative care needs and palliative care satisfaction (*N* = 114)

Disease type	Total (*N* = 114)	CHF ^a^ (*n* = 35)	Stroke ^b^ (*n* = 21)	ESRD ^c^ (*n* = 28)	ESLD ^d^ (*n* = 30)	*F*	*p*	Post-hoc (Scheffé)
Variables	*M* ± *SD*	*M* ± *SD*	*M* ± *SD*	*M* ± *SD*	*M* ± *SD*			
Palliative care needs								
Physical	3.70 ± 0.72	3.95 ± 0.74	3.36 ± 0.69	3.68 ± 0.64	3.66 ± 0.73	3.114	0.029	a > b
Psychosocial	3.83 ± 0.67	3.95 ± 0.65	3.61 ± 0.84	3.74 ± 0.58	3.92 ± 0.61	1.469	0.227	
Spiritual	3.23 ± 0.88	3.50 ± 0.93	3.13 ± 0.83	3.10 ± 0.85	3.15 ± 0.86	1.491	0.221	
Educational/referral	3.81 ± 0.73	3.91 ± 0.79	3.54 ± 0.84	3.83 ± 0.68	3.89 ± 0.60	1.288	0.282	
Total	3.66 ± 0.62	3.84 ± 0.67	3.42 ± 0.72	3.60 ± 0.51	3.68 ± 0.53	2.197	0.092	
Palliative care satisfaction								
Physical	3.46 ± 0.65	3.66 ± 0.60	3.28 ± 0.73	3.31 ± 0.65	3.48 ± 0.60	2.320	0.079	
Psychosocial	3.44 ± 0.71	3.64 ± 0.71	3.27 ± 0.84	3.29 ± 0.61	3.47 ± 0.69	1.804	0.151	
Spiritual	2.85 ± 0.90	2.78 ± 0.95	2.87 ± 0.80	3.10 ± 0.58	2.70 ± 1.13	1.050	0.374	
Educational/referral	2.97 ± 0.80	2.83 ± 0.90	2.97 ± 0.76	3.21 ± 0.58	2.90 ± 0.85	1.344	0.264	
Total	3.60 ± 0.56	3.76 ± 0.57	3.40 ± 0.72	3.51 ± 0.44	3.63 ± 0.49	2.257	0.086	

a, b, c, d  group for Scheffé tests, *M *mean, *SD *standard deviation, *CHF *chronic heart failure, *ESRD *end-stage renal disease, *ESLD *end-stage liver disease

### Anxiety and depression

Table 3 shows the levels of participants’ anxiety and depression. The average anxiety score of the participants was 7.48 ± 3.60 (range: 0–21). Most participants (50.0%) had normal levels of anxiety (range: 0–7), followed by borderline cases (32.5%; range: 8–10) and abnormal cases (17.5%; range: 11–21). The average depression score of the participants was higher than anxiety at 9.17 ± 3.71 (range: 0–21). Abnormal levels of depression (range: 11–21) accounted for the largest share (37.2%), followed by normal cases (31.9%; range: 0–7) and borderline cases (31.0%; range: 8–10). There were no significant differences in the levels of anxiety and depression by disease type.

**Table 3:** Level of anxiety and depression (*N = 114*)

Disease type	Total (*N* = 114)	CHF (*n* = 35)	Stroke (*n* = 21)	ESRD (*n* = 28)	ESLD (*n* = 30)	*F* or χ^2^*(p)*
Variables	*M* ± *SD*/*n* (%)	*M* ± *SD*/*n* (%)	*M* ± *SD*/*n* (%)	*M* ± *SD*/*n* (%)	*M* ± *SD*/*n* (%)	
Anxiety (0–21)	7.48 ± 3.60	7.40 ± 3.29	7.14 ± 4.05	7.36 ± 3.81	7.91 ± 3.56	0.216 (0.885)
Normal (0–7)	57 (50.0)	15 (42.9)	13 (61.9)	12 (42.9)	17 (56.7)	8.029 (0.241)
Borderline (8–10)	37 (32.5)	16 (45.7)	3 (14.3)	11(39.3)	7 (23.3)	
Abnormal (11–21)	20 (17.5)	4 (11.4)	5 (23.8)	5 (17.9)	6 (20.0)	
Depression (0–21)	9.17 ± 3.71	9.17 ± 3.71	9.43 ± 3.61	9.57 ± 3.63	8.62 ± 3.54	0.359 (0.783)
Normal (0–7)	36 (31.9)	8 (22.9)	6 (28.6)	9 (32.1)	13 (44.8)	5.949 (0.438)
Borderline (8–10)	35 (31.0)	15 (42.9)	5 (23.8)	8 (28.6)	7 (24.1)	
Abnormal (11–21)	42 (37.2)	12 (34.3)	10 (47.6)	11 (39.3)	9 (31.0)	

*M *mean, *SD *standard deviation, *CHF *chronic heart failure, *ESRD *end-stage renal disease, *ESLD *end-stage liver disease

### Health-related quality of life

Participants’ HRQOL is shown in Table 4. The average score of PCS, indicating physical HRQOL, was 38.89 ± 8.69. The average PCS score for the stroke group was 42.39 ± 7.79, which was significantly higher than the CHF group with 36.11 ± 7.92 (*F* = 3.722, *p* = .014). Among the four domains, the role-physical score of the stroke group was 44.43 ± 8.91, which was significantly higher than the CHF (35.73 ± 10.40) and ESRD (33.74 ± 9.22) groups (*F* = 5.830, *p* = .001). The average MCS score, indicating mental HRQOL, was 40.43 ± 11.19. Although the average MCS score for the stroke group was higher at 45.30 ± 11.06, there was no significant difference by disease type. Among the four domains, the vitality score of the stroke group was 46.09 ± 9.66, which was significantly higher than the CHF (37.74 ± 10.90) and ESRD (38.17 ± 10.53) groups (*F* = 3.600, *p* = .016).

**Table 4:** Level of the health-related quality of life (*N = 114*)

Disease type	Total (*N* = 114)	CHF ^a^ (*n* = 35)	Stroke ^b^ (*n* = 21)	ESRD ^c^ (*n* = 28)	ESLD ^d^ (*n* = 30)	*F (p)*	Post-hoc (Scheffé)
Variables	*M* ± *SD*	*M* ± *SD*	*M* ± *SD*	*M* ± *SD*	*M* ± *SD*		
Physical component scale	38.89 ± 8.69	36.11 ± 7.92	42.39 ± 7.79	37.16 ± 9.60	41.29 ± 8.11	3.722 (0.014)	b > a
Physical functioning	38.52 ± 10.38	36.54 ± 9.51	41.14 ± 10.54	35.46 ± 11.01	41.85 ± 9.71	2.837 (0.041)	
Role-physical	37.89 ± 10.30	35.73 ± 10.40	44.43 ± 8.91	33.74 ± 9.22	39.72 ± 9.81	5.830 (0.001)	b > a, c
Bodily pain	39.39 ± 10.98	34.67 ± 9.54	42.20 ± 10.89	40.86 ± 11.50	41.56 ± 10.92	3.365 (0.021)	
General health	38.42 ± 10.05	37.37 ± 9.20	42.82 ± 10.73	36.14 ± 10.41	38.68 ± 9.67	2.008 (0.117)	
Mental component scale	40.43 ± 11.19	39.48 ± 11.31	45.30 ± 11.06	37.57 ± 11.41	40.80 ± 10.28	2.095 (0.105)	
Vitality	40.51 ± 10.63	37.74 ± 10.90	46.09 ± 9.66	38.17 ± 10.53	42.00 ± 9.70	3.600 (0.016)	b > a, c
Social functioning	39.93 ± 11.34	39.72 ± 10.97	42.78 ± 11.75	36.93 ± 12.50	40.96 ± 10.19	1.187 (0.318)	
Role-emotional	36.84 ± 12.43	36.27 ± 12.25	43.07 ± 11.06	34.03 ± 12.51	35.74 ± 12.59	2.425 (0.069)	
Mental health	41.23 ± 11.63	38.76 ± 12.22	45.01 ± 12.38	38.35 ± 10.71	44.15 ± 10.23	2.576 (0.057)	

a, b, c, d group for Scheffé tests, *M *mean, *SD *standard deviation, *CHF *chronic heart failure, *ESRD *end-stage renal disease, *ESLD *end-stage liver disease

### Health-related quality of life according to participants’ demographic and clinical characteristics

Among the two dimensions of HRQOL, PCS scores differed significantly according to sex (*t* = 2.145, *p* = .034), employment status (*t* = 2.085, *p* = .039), duration of illness (*t* = 2.085, *p* = .040), and ECOG PS (*F* = 6.722, *p* = .002). PCS scores were lower among women, the unemployed, and those with a duration of illness for 12 months or longer. With regards to ECOG PS, the PCS score of the Grade 2 participants was significantly lower than that of Grades 0 and 1. MCS, the other dimension of HRQOL, showed a significant difference according to ECOG PS (*F* = 4.705, *p* = .011); it was lower for ECOG PS Grade 2 patients than Grades 0 and 1.

Concerning HRQOL differences within disease-type groups, in the case of the CHF group, the PCS score of the New York Heart Association (NYHA) class III and IV participants was significantly lower than that of NYHA class I and II participants (*t* = 2.882, *p* = .007), and the score of participants with an ejection fraction of 50% or less was lower than that of participants with 50% or more (*t* = 2.310, *p* = .027). There was no significant difference according to the clinical characteristics of the CHF group in MCS. In the stroke group, there was a significant negative correlation between the participants’ activities of daily living levels and PCS (*r* = − .637, *p* = .002). There was a significant positive correlation between serum albumin levels and PCS in the ESLD group (*r* = .455, *p* = .012).

### Correlations among the main variables

We analysed the correlations among palliative care needs, palliative care satisfaction, anxiety, depression, HRQOL, and participants’ general characteristics. Participants’ PCS had a significant negative correlation with duration of hospitalisation (*r* = − .268, *p* = .004) and the physical domains of palliative care needs (*r* = − .239, *p* = .010). In the case of MCS, there was a significant negative correlation with the physical domains of palliative care needs (*r* = − .303, *p* = .001), the psychosocial domains of palliative care needs (*r* = − .283, *p* = .002), anxiety (*r* = − .406, *p* < .001), and depression (*r* = − .345, *p* < .001).

### Factors influencing health-related quality of life

Multiple linear regression analysis was performed on PCS (indicating physical HRQOL) and MCS (indicating mental HRQOL) to identify factors influencing the HRQOL of participants (Table 5). Multiple linear regression analysis included variables with a significance level of *p* < .05 from the univariate and bivariate analysis: in the case of PCS, the significant independent variables were sex, employment status, duration of illness, and ECOG PS; in the case of MCS, the physical and psychosocial domains of palliative care needs, anxiety, and depression were included.

We found that the duration of hospitalisation and ECOG PS were significant factors for PCS. Increasing the duration of hospitalisation was associated with lower physical HRQOL (*β* = − 0.222, *p* = .012). Participants with ECOG PS Grade 2 displayed lower physical HRQOL than those with ECOG Grades 0 and 1 (*β* = -4.599, *p* = .022). Further, in the case of factors for MCS, ECOG PS, type of disease, anxiety, and depression were significant. Participants with ECOG PS Grade 2 displayed lower mental HRQOL than those with ECOG Grades 0 and 1 (*β* = -5.794, *p* = .017). The ESRD group displayed lower mental HRQOL than the stroke group (*β* = -7.318, *p* = .011). High anxiety (*β* = − 0.721, *p* = .013) and depression (*β* = − 0.714, *p* = .013) scores were associated with lower mental HRQOL.

**Table 5:** Factors influencing health-related quality of life *(N = 114)*

Independent variables	PCS			MCS		
	β	*S.E.*	*t (p)*	β	*S.E.*	*t (p)*
Sex (Women)	-1.540	1.724	0.893 (0.374)			NA
Employed (Yes)	0.244	1.744	0.140 (0.889)			NA
Duration of disease (≥ 12 months)	-2.724	1.704	0.148 (0.113)			NA
Duration of hospitalisation	-0.222	0.086	0.086 (0.012)			NA
ECOG PS 2 (Ref = 0, 1)	-4.599	1.979	2.324 (0.022)	-5.794	2.391	2.423 (0.017)
ECOG PS 3, 4 (Ref = 0, 1)	-1.720	2.141	0.080 (0.424)	0.378	2.610	0.145 (0.885)
CHF (Ref = Stroke)	-2.893	2.402	1.204 (0.231)	-3.328	2.737	1.216 (0.227)
ESRD (Ref = Stroke)	-3.305	2.371	1.394 (0.166)	-7.318	2.821	2.594 (0.011)
ESLD (Ref = Stroke)	1.469	2.477	0.593 (0.555)	-3.692	2.761	1.337 (0.184)
Physical palliative care needs	-1.560	1.076	0.803 (0.424)	-1.428	1.750	0.816 (0.416)
Psychosocial palliative care needs			NA	-2.261	1.864	1.213 (0.228)
Anxiety			NA	-0.721	0.286	2.520 (0.013)
Depression			NA	-0.714	0.284	2.517 (0.013)

VIF: PCS 1.078–2.306; MCS 1.173–2.029

*PCS* Physical Component Summary, *MCS *Mental Component Summary, *CHF *chronic heart failure, *ESRD *end-stage renal disease, *ESLD *end-stage liver disease, *ECOG PS *Eastern Cooperative Oncology Group Performance Status, *NA *not applicable

## Discussion

This study examined the palliative care needs and satisfaction, anxiety and depression, and HRQOL in patients with common non-cancer diseases and identified which factors influence their HRQOL.

### Palliative care needs and satisfaction

Participants’ palliative care needs score was 3.66 ± 0.62, which was similar to that of patients with terminal cancer (3.58 ± 0.31) admitted to a hospice ward (using the same measurement tool) [32]. Palliative care needs levels in the physical and spiritual domain of non-cancer patients in this study were similar to those of patients with cancer, while the psychosocial and educational/referral domains were higher. In this study, the importance of palliative care need domains was ranked in the following descending order: psychosocial, educational/referral, physical, and spiritual. This differed from patients with cancer, among whom the order was: physical, educational/referral, psychosocial, and spiritual [32]. The results showed that participants’ psychosocial and educational/referral needs were higher than those of patients with cancer, indicating that they had different priorities. In another study that analysed electronic medical records retrospectively, non-cancer patients complained of dyspnoea more commonly than patients with cancer and their palliative care needs in psychosocial aspects such as anxiety, depression, and concerns were higher than those of patients with cancer [8]. In a systematic review on the prevalence of palliative care-related problems, although there were commonalities in the results of high problem prevalence in the physical and psychosocial domains of patients with cancer and non-cancer patients [41], there were differences in the types and frequencies of symptoms. This supports the need for a palliative care model that considers the specific palliative care needs of non-cancer patients, rather than merely applying the palliative care model developed for patients with cancer. This coincides with a previous study comparing patients with cancer and non-cancer patients (ESRD, heart failure, and COPD), which reported that the functional status of non-cancer patients should be paid attention to and that they require more comprehensive care, even if they go through a disease progression similar to that of patients with cancer [9]. Although the survival rate of patients with COPD is higher than that of patients with lung cancer, COPD patients have a similar level of symptom burden and palliative care needs [42]. It has also been shown that ESRD patients experience various symptoms that relate to their physical, psychological, social, existential, and practical needs [43]. Hence, even in the case of terminal non-cancer patients, there is a need for palliative care in various domains. This integrated palliative care should include psychosocial, spiritual, and educational/referral aspects while maintaining the provision of palliative care in the physical domain for alleviation of physical symptoms and enhancement of non-cancer patients’ functional status.

In this study, the psychosocial domain of the palliative care needs was higher than others. Because physical symptoms (e.g. dyspnoea, nutritional problems, etc.) tend to be prioritised and care for them has already been met to some extent, non-cancer patients may have reported relatively high psychosocial needs compared to other domains of palliative care. Contrastingly, it has been considered in oncology care that early assessment and management of the psychosocial problem is important from the diagnosis and the beginning of treatment [44, 45]. In addition, the National Comprehensive Cancer Network (2022) recommends identifying physical, emotional, social, practical, spiritual, and religious concerns by an integrative approach using the Problem List in Distress Thermometer and evaluating and managing according to said guidelines. Therefore, we need to try to meet the psychosocial palliative care needs of non-cancer patients in practice, just as dealing with managing psychosocial problems is essential in oncology care. However, although there is no significant difference in palliative care needs according to disease type, the total score of palliative care needs in the CHF group was higher than that in the stroke, ESRD, and ESLD groups. This could be owing to participants’ clinical characteristics, as CHF patients had the highest number of comorbidities and the longest duration of illness among the four disease groups. Patients’ unmet needs require consideration in early palliative care before they worsen.

Participants’ palliative care satisfaction level was similar to that of patients with terminal cancer admitted to a hospice ward [32]. However, they had lower levels of palliative care satisfaction than palliative care needs, as with patients with cancer [32]. Studies have been reported consistently high palliative care satisfaction in bereaved caregivers of cancer and non-cancer patients who received palliative care [16, 46]. However, since it was not obtained from the patients, the main beneficiary of palliative care, it is difficult to determine how much the patients’ palliative care needs were met. In addition, although it was reported that palliative care improved patients’ satisfaction with a small effect size [47], there was a limitation in the lack of studies on the non-cancer patient population. Considering the associations between low satisfaction and higher hospital costs [16], it is necessary to continuously evaluate and improve palliative care satisfaction from patients’ perspectives.

### Anxiety and depression

The average anxiety score of participants in this study was 7.48 ± 3.60 and depression was 9.17 ± 3.71. Among participants, 50% had anxiety symptoms (HADS-A ≥ 8), and 68.2% had depression symptoms (HADS-D ≥ 8). Participants’ anxiety and depression levels were higher than those among patients with cancer: anxiety (ranged 5–6.1) and depression scores (ranged 4.5–7.2) [48, 49], and were similar to those of patients with terminal cancer with less than six months of life expectancy [50]. In studies on non-cancer patients, stroke patients scored 7.5 and 6.5, for anxiety and depression, respectively [51]; and ESLD patients scored 6.7 and 5.5, respectively [52], which were lower than the current results. The proportion of patients with anxiety and depression in this study was also higher than that of patients with cancer and non-cancer patients in previous studies [53, 54]. We found that non-cancer patients had similar or greater prevalence of severe emotional health problems to patients with cancer from previous studies. Perhaps anxiety and depression in patients with cancer are managed to some extent by applying the standard that provides early assessment and appropriate interventions regarding psychosocial symptoms. Therefore, special attention should be paid to screening for anxiety and depression as they increase suffering among patients experiencing various physical symptoms [42]. In addition, it is necessary not to overlook the psychological symptoms of non-cancer patients, and palliative care should be provided considering each patient’s degree of anxiety and depression.

In this study, participants’ depression scores were higher than their anxiety scores. It might be due to the clinical characteristics of patients. Anxiety scores are expected to be higher than depression in the initial stage of the disease. However, most participants in this study were in the end-stage that had progressed for a long time. Hence, it might have resulted in higher scores in the depression subscale consisting of questions such as the loss of pleasure and low mood than in the anxiety subscale. We found no consistency in the trends regarding scores and rates of anxiety and depression in previous studies. Haemodialysis patients had slightly higher scores and rates of depression than anxiety at the baseline and after one year [55]. A longitudinal study evaluating anxiety and depression in stroke patients for three years reported that both anxiety and depression deteriorated over time; however, the scores and rates of anxiety were higher than those of depression at the final follow-up [56]. In patients with cancer, the anxiety score was higher than that of depression, and the prevalence of anxiety and depression was higher after 5–6 years than at the time of diagnosis [57]. Therefore, it is necessary to regularly assess anxiety and depression from the first diagnosis of non-cancer diseases and to identify trajectories over time.

### Health-related quality of life

Physical HRQOL was scored 38.89 ± 8.69 and mental HRQOL was scored 40.43 ± 11.19. The physical HRQOL of the participants in this study was similar to or higher than that of patients with cancer measured using the same tool previously, but their mental HRQOL was lower [58, 59]. In the domains of HRQOL, the role-physical domain was the lowest with 37.89 ± 10.30 in physical HRQOL and the role-emotional domain was the lowest with 36.84 ± 12.43 for mental HRQOL. These results are similar to those of a study of patients with uncommon cancers; moreover, enhancing the role-physical and role-emotional domains may be effective in improving the HRQOL of patients [58]. The HRQOL of the participants in this study was similar to that of non-cancer patients in other studies [60, 61]. Even if a patient faces physical limitations owing to disease, it is necessary to apply palliative care that can increase their physical HRQOL by improving physical function to the greatest extent possible. In addition, as shown in the results of palliative care needs, anxiety, and depression, attention must be paid to mental healthcare for non-cancer patients, and palliative care should focus on improving mental HRQOL.

Physical HRQOL in stroke group was higher than other disease groups, especially, significantly higher than CHF group. The reasons for the higher physical HRQOL of stroke patients than others are as follows. Owing to the characteristic of the tertiary hospital where this study was conducted, there are more stroke patients requiring acute treatment for sudden attack than patients requiring palliative care. For these patients, the goal of treatment is to recover normal functioning rather than discuss palliative care. In addition, patients with relatively good physical functioning participated in this study since it is difficult for patients with reduced consciousness, language ability, or physical movement to respond to the questionnaire. This demonstrates that it is necessary to investigate the palliative care needs, mental health status, and HRQOL of stroke patients admitted to small and medium hospitals or long-term facilities.

### Factors influencing health-related quality of life

The factors influencing the physical HRQOL of non-cancer patients were the length of hospitalisation and ECOG PS, showing a similar tendency to the results of a previous study on end-stage COPD and patients with lung cancer [62]. Even with worse ECOG PS, negative changes in QOL may be reduced if the patient’s health status allows for the preservation of physical functions and performance of activities of daily living. Therefore, it is necessary to predict the QOL of non-cancer patients through physical function assessment. Furthermore, recognising that deterioration in physical function affects both physical and mental HRQOL, the development of tailored palliative care nursing services considering the condition of non-cancer patients is required.

The factors influencing mental HRQOL were anxiety and depression. Previous studies also reported that anxiety and depression affect HRQOL [48, 63, 64]. However, in practice, the management of disease-related physical symptoms of non-cancer patients receives more attention, and the assessment and management of mental health tend to be less emphasised. In addition, pharmacological interventions tend to be prioritised for mental health problems in terminally ill patients. It is important to detect mental health problems such as anxiety and depression early and manage them in advance to prevent deterioration of mental HRQOL.

In conclusion, we believe that medical staff can find modifiable factors among several that can be considered as influencing factors on the HRQOL and used to improve the QOL. Even if disease-related factors (e.g. duration of illness) cannot be modifiable, maintaining patients’ best physical function within possibility could be helpful to improve physical-related QOL. The level of psychological problems that influence mental HRQOL can be modified by providing regular assessment, follow-up, and appropriate management from the time of diagnosis of non-cancer patients. Therefore, it is necessary to develop practical guidelines and educate medical staff to provide early palliative care in other units as well as the palliative care ward.

### Limitations

This study had some limitations. First, the participants were patients who were admitted to a tertiary hospital, and since most of the stroke patients recovered after acute treatment, the HRQOL of stroke patients tends to be measured more positively compared to other disease groups. Hence, the results should be interpreted carefully. Second, since our participants had little experience with palliative care, caution is required when interpreting the results of palliative care satisfaction. Third, in the case of participants with worse ECOG PS, there was a limited identification of their QOL; recruiting these participants was difficult owing to their poor health conditions. Finally, there is a limitation related to the validity of the palliative care needs and satisfaction questionnaire. Therefore, we suggest that further research with a large sample of non-cancer patients is needed to verify the scale’s content validity.

Despite these limitations, this study is meaningful in that it provides basic data for the development of a South Korean palliative care model by expanding the understanding of palliative care needs and examining HRQOL in non-cancer patients. In addition, even though the South Korean government implemented the Act on Hospice and Palliative Care and Decisions on Life-Sustaining Treatment for Patients at the End of Life in accordance with international trends, and non-cancer patients are now included as participants of palliative care, preparations for applying palliative care to non-cancer patients are insufficient in practice. This study is meaningful in that it focuses on patients who are relevant to the emerging need to consider palliative care for terminally ill non-cancer patients in South Korea.

## Conclusion

We found that the palliative care needs and satisfaction, anxiety and depression, and HRQOL of non-cancer patients were similar to those of patients with cancer, and their physical function and mental health were connected with their HRQOL. Therefore, it is necessary to maintain the level of patients’ physical ability and to assess and manage mental health in advance for non-cancer patients in palliative care. Furthermore, it is required to develop a tailored palliative care model for non-cancer patients that reflects their palliative care needs, physical performance status, and psychological status.

## Data Availability

The datasets generated and analysed during the current study are not publicly available owing to institutional policy but are available from the corresponding author on reasonable request.

## Abbreviations

CHF: Chronic heart failure
COPD: chronic obstructive respiratory disease
ECOG PS: Eastern Cooperative Oncology Group Performance Status
ESLD: end-stage liver disease
ESRD: end-stage renal disease
HRQOL: health-related quality of life
HADS: Hospital Anxiety and Depression Scale
MOS SF-36: Medical Outcome Study 36-items Short Form Health Survey
MCS: Mental Component Summary
NYHA: New York Heart Association
PCS: Physical Component Summary
QOL: Quality of life
